# Effect of alkaline lignin modification on cellulase–lignin interactions and enzymatic saccharification yield

**DOI:** 10.1186/s13068-018-1217-6

**Published:** 2018-08-01

**Authors:** Wenjun Ying, Zhengjun Shi, Haiyan Yang, Gaofeng Xu, Zhifeng Zheng, Jing Yang

**Affiliations:** 1 Key Laboratory for Forest Resources Conservation and Utilization in the Southwest Mountains of China, Ministry of Education, Southwest Forestry University, Kunming, 650224 China; 2 School of Chemical Engineering, Southwest Forestry University, Kunming, 650224 China

**Keywords:** Lignocellulosic biomass, Biomass pretreatment, Lignin modification, Nonproductive adsorption, Enzymatic saccharification

## Abstract

**Background:**

The lignin can compete for binding cellulase enzymes with cellulose fibers and decrease the accessibility of enzymes to carbohydrates. The competitive adsorption of cellulase to lignin mainly depended on the chemical structure of lignin. The post-pretreatment can decrease the lignin content and modify the lignin structure of pretreated substrates, which reduced the lignin inhibition on enzymatic saccharification. Therefore, the post-treatment by modifying the lignin structure would attract considerable attention for weakening the cellulase–lignin interactions.

**Results:**

Three modified lignins, including sulfonated lignin (SL), oxidized lignin (OL), and carboxylated lignin (CL), were prepared from alkali lignin (AL) and their structures and physicochemical properties were characterized using FTIR, NMR, XPS analysis, zeta potential, and contact angle, respectively. Compared to AL, three modified lignin preparations exhibited the decrease in contact angle by 61–70% and phenolic hydroxyls content by 17–80%, and an obvious increase of negative charges by about 21–45%. This was mainly due to the drop of condensation degree and the incorporation of carboxylic and sulfonic acid groups into modified lignins. Langmuir adsorption isotherms showed that the affinity strength between cellulase and modified lignins significantly reduced by 54–80%. Therefore, the 72 h hydrolysis yield of Avicel with SL, OL, and CL was 48.5, 51.3, and 49.4%, respectively, which was increased 8–15.3% than that of Avicel with AL, 44.5%. In the enzymatic hydrolysis of bamboo biomass, the glucose yield at 5 d was 38.5% for AS-*P. amarus*, 15.4% for AO-*P. amarus* and 21.4% for AC-*P. amarus*, respectively, which were 1.4–3.5 times of alkali pretreated *P. amarus*.

**Conclusions:**

The post-treatment can weaken the nonproductive adsorption between lignin and cellulase proteins and improve the enzymatic saccharification efficiency. This study will provide a conceptual combination of pretreatment technologies and post-pretreatment by modifying lignin structure for reducing the cellulase–lignin interaction.

## Background

Biochemical conversion of lignocellulosic biomass to sugar-based compounds has been devoted for the production of biofuels and platform chemicals such as 5-hydroxymethylfurfural and furfural [1]. A crucial commercialization-limiting step in the processes is the enzymatic hydrolysis of polysaccharides in lignocellulose to simple sugars [2]. The factors affecting enzymatic hydrolysis of lignocellulosic biomass includes substrate-related factors, mainly consisting of the presence of lignin and hemicellulose in biomass, the degree of polymerization and crystallinity of cellulose, the surface area accessible to cellulases, and enzyme-related factors [3, 4]. Amongst factors, lignin plays an inhibitory role in the enzymatic digestibility of polysaccharides in plant cell walls.

Lignin is a cross-linked polymer and consists of different phenylpropane, such as *p*-hydroxyphenyl (H), guaiacyl (G), and syringyl (S) units, through the linkage of various interunit linkages, such as β-*O*-4′, β-5′, β-β′, 5-5′, 5-*O*-4′, and β-1′ [5, 6]. In addition, lignin contains plentiful functional groups, such as aliphatic hydroxyl, phenolic hydroxyl, carbonyl, carboxyl, and methyl groups [6]. Therefore, lignin can not only form a shield to prevent carbohydrate from the enzyme attack [7], but also adsorb cellulase enzymes nonproductively and irreversibly via hydrophobic, electrostatic, and hydrogen bond interactions, making it undesirable for enzymatic digestion of biomass [8, 9]. Although the lignin shield can be disrupted by the biomass pretreatment and lignin can be usually partially removed, a considerable part of residual lignins still remain in the pretreated substrates. And the complete removal of lignin, the term “delignification”, is costly and difficult for the existing thermochemical pretreatment technologies. As a result, the nonproductive binding between lignin and cellulase enzymes was unavoidable and led to the decrease of glucose release in the enzymatic hydrolysis process.

How to reduce the cellulase–lignin interaction for enhancing the enzymatic saccharification efficiency has been widely studied. The anionic surfactant and hydrophobic proteins, as lignin blocker, had been reported to be effective to weaken or eliminate the nonproductive adsorption by blocking the exposed sites of lignin surfaces, thereby decreasing the cellulase activity loss [10, 11]. Lignosulfonate (LS) can improve the cellulolytic hydrolysis efficiency due to the increase of negative charges, and further lead to the increase of free enzyme content in supernatant [12]. Certain metal ions, such as Ca^2+^ and Mg^2+^, might also occupy the active sites of lignin and form lignin–metal complex, which blocked the interaction between cellulase and lignin [13, 14]. The free phenolic hydroxyls in lignin matrix can decrease enzymatic hydrolysis efficiency of lignocellulose by forming hydrogen bonding with cellulase enzymes, but the inhibitory effect could be removed by lignin hydroxypropylation [15, 16]. It had also found that the presence of carboxylic groups in lignin might reduce the negative effects of lignin on enzymatic hydrolysis of pretreated substrates by increasing hydrophilicity and electrostatic repulsion against enzymes [17]. Lignin S/G ratios may significantly influence sugar release of lignocellulosic biomass [18, 19]. In addition, genetic engineering had been explored to modify the pathways of lignin biosynthesis to decrease lignin content and alter the relative proportions of lignin subunit (G, S, and H units) [20–22]. These evidences clearly indicated that the nonproductive binding of enzymes to lignin can greatly attribute to the interaction between lignin functional groups and the cellulase protein.

The primary goal of this work is to modify the surface properties and structure of residual lignin through introducing functional groups, which have potential for reducing the cellulase–lignin interaction. Modified lignin preparations, including sulfonated lignin (SL), oxidized lignin (OL) and carboxylic lignin (CL), were prepared from alkali lignin (AL), and their structural properties were thoroughly investigated by FTIR, 13C, 2D HSQC NMR, and XPS analysis. The phenolic hydroxyl group content, the amount of negative charges and contact angle of lignin preparations were measured, respectively. The Langmuir adsorption isotherm was used to characterize enzyme affinity to lignin preparations. Finally, the effect of modified lignins on the enzymatic digestion of cellulose were evaluated and compared; whether the lignin modification as post-treatments can work on lignocellulosic biomass was also investigated.

## Results and discussions

### Structural characteristic of lignin preparations

**Fig. 1:**
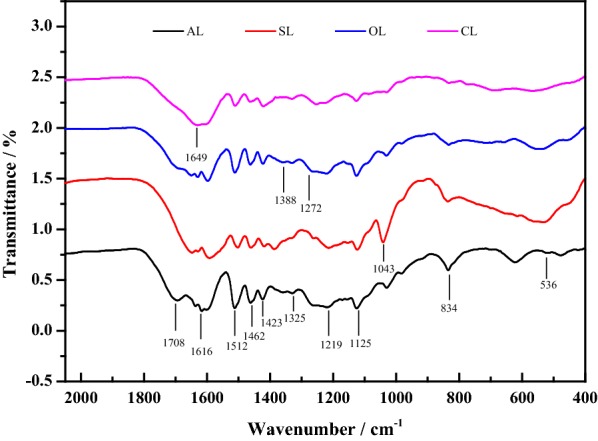
FTIR spectra of lignin samples. Alkali lignin, sulfonated lignin, oxidized lignin, and carboxylated lignin were expressed as AL, SL, OL, and CL, respectively

The FTIR, ^13^C and 2D HSQC NMR were used to characterize the chemical structure of lignin preparations. The fingerprint region (800–1800 cm^−1^) of FTIR spectra, corresponding to the stretching vibrations of different groups in lignin preparations, is displayed in Fig. 1, and the bands are assigned in Table 1 based on the previous studies [23, 24]. As shown in Fig. 1, obvious characteristic peaks at 1616, 1512, and 1423 cm^−1^ observed in all samples were the aromatic skeletal vibrations, implying that the core of lignin structure was not altered significantly during the modified process. However, the spectra also showed some changes in the peaks and the absorption intensities. The peak at 1708 cm^−1^ characteristic of non-conjugated carbonyl groups is much more intense in AL than OL. And even such a band was not present in SL and CL. It could be due to the carboxyl or ester linkage of C_γ_ in AL side chain was partially cleaved during the modification process. The absorption band of 1649 cm^−1^, assigned to conjugated carbonyl groups, was identified in SL, OL, and CL. The intensity of 1272 cm^−1^ corresponding to the C–O stretching of G type lignin had not been significantly changed after modifications. The signal intensity of *p*-hydroxyphenyl units (H unit) at 834 cm^−1^ was reduced in modified lignin, compared with that of AL, indicating the dissolving of H-type lignin. As compared to AL, OL, and CL, the successful introduction of the sulfonic groups into SL can be justified by the stronger bands at 1043 cm^−1^ (S=O stretching vibration) and 536 cm^−1^ (C–S stretching vibration).

**Table 1:** Assignments of FTIR peaks in lignin samples [23, 24]

Wavenumber/cm^−1^	Assignments
1616, 1512, 1423	Aromatic skeletal vibrations
1708	Non-conjugated carbonyl groups
1649	Conjugated carbonyl groups
1272	The C–O stretching of G type lignin
1043	S=O stretching vibration
834	*p*-hydroxyphenyl units (H unit)
536	C–S stretching vibration

**Fig. 2:**
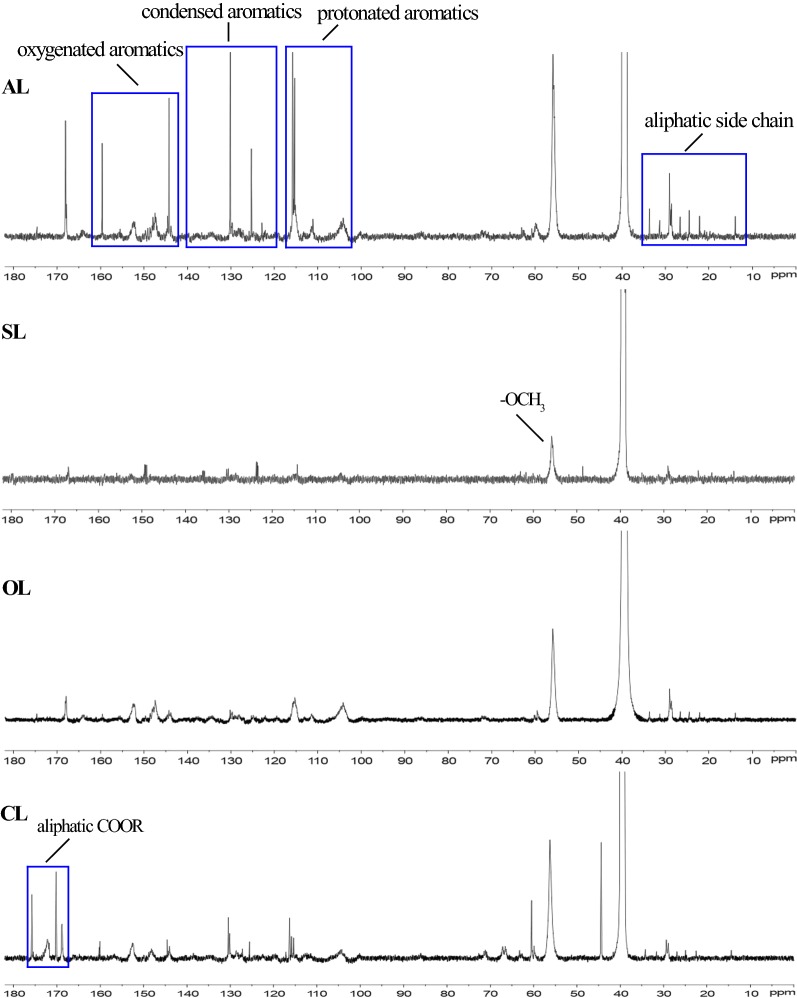
^13^C NMR spectra of lignin samples

In the ^13^C NMR spectra (Fig. 2) and the assignment of correlation signals (Table 2) of lignin, the aromatic structure region (103–162 ppm) was easily identified by correlation signal at 110.6, 115.6, 119, 103, and 152.3 ppm, respectively, corresponding to G_2_, G_5_, G_6_, S_2,6_, and S_3,5_ positions [25, 26]. As shown in Fig. 2, the signal intensities of S_3,5_ units and S_2,6_ in three modified lignins were greatly decreased compared to those of AL, and the intensities of G_2_, G_5_, and G_6_ units also showed a similar trend with S units. The *p*-hydroxyphenyl (H) units occurred as two signals at 128.0 and 129.2 ppm (C-2/C-6); and the lignin sulfonation, oxidation and carboxylation can reduce the level of H-lignin unit. The transformation of above signals indicated that lignin polymer was dissociated and the modification can improve the hydrophilicity and solubility of lignin due to altering the relative proportions of lignin subunit (G, S, and H units). Moreover, the bands of condensed aromatics (140–123 ppm) became weak and narrow in SL, OL, and CL compared to those in AL, implying that fewer condensation reactions could occur between free lignin units during the modification. The signal intensities between 50 and 90 ppm corresponding to lignin interunit linkages had no changes, suggested that the modification could occur on the surface of lignin moieties. The decreasing signal intensities for –OCH_3_ can be seen at 56.5 ppm, implying that the modification reactions could cause demethoxylation and demethylation in AL. In addition, the aliphatic COOR at 178–168 ppm are more abundant in CL, followed by OL and SL. In the carboxylation reaction, the H atom in lignin hydroxyl was substituted by the –OCH_2_COOR, which could be responsible for the most abundant aliphatic COOR.

**Table 2:** Signal assignments of lignin samples in the ^13^C NMR spectrum [25, 26]

*δ* (ppm)	Assignments	*δ* (ppm)	Assignments
178-168	Aliphatic COOR	115.6	C_5_ in G unit
152.3	C_3_/C_5_ in S unit	110.6	C_2_ in G unit
140-123	Condensed aromatics	103	C_2,6_ in S unit
129.2	C_6_ in H unit	50–90	Interunit linkages
128	C_2_/C_6_ H unit		(β-β′, β-5 and β-*O*-4′)
119	C_6_/C_5_ in G unit	56.5	OCH_3_ in G and S unit

G: guaiacyl unit, S: syringyl unit, H: *p*-hydroxyphenyl unit

To further structural investigation, the 2D HSQC NMR was used to analyze the structural units and different interunit linkages of lignin polymers. The HSQC spectra consisted of the side chain (*δ*_C_/*δ*_H_ 50–95/2.5–6.0) and aromatic regions (*δ*_C_/*δ*_H_ 95–160/5.5–8.50) and correlation signals are assigned in Table 3 according to previous publications [26, 27].

**Table 3:** Assignments of ^13^C-^1^H correlation signals in the HSQC spectrum of lignin samples [26, 27]

Signals	*δ*_C_/*δ*_H_	Assignments
MeO	55.4/3.72	C-H in methoxyls
*A*_*γ*_	59.5–59.7/3.40–3.63	C_*γ*_-H_*γ*_ in *β*-*O*-4′ substructures (A)
*A*_*α*_	71.6/4.88	C_*α*_-H_*α*_ in *β*-*O*-4′ substructures linked to a S unit (A)
*A*_*β*(G)_, *A*_*β*(S)_	82.8–85.7/4.13–4.46	C_*β*_-H_*β*_ in *β*-*O*-4′ substructures linked to a G unit (A) C_*β*_-H_*β*_ in *β*-*O*-4′ substructures linked to a S unit (A)
S_2, 6_	103.8/6.69	C_2,6_-H_2,6_ in etherified syringyl units (S)
S′_2, 6_	106.4/7.31	C_2, 6_-H_2, 6_ in oxidized (C_*α*_= O) phenolic syringyl units (S′)
G_5_	115.1/6.72	C_5_-H_5_ in guaiacyl units (G)
G_2_	111.0/7.02	C_2_-H_2_ in guaiacyl units (G)
G_6_	119.0/6.77	C_6_-H_6_ in guaiacyl units (G)
FA_2_	110.8/7.35	C_2_-H_2_ in FA
FA_6_	123.1/7.16	C_6_-H_6_ in FA
FA_7_	144.4/7.42	C_7_-H_7_ in FA
H_2, 6_ *p*CA	127.5/7.23 129.8/7.52 143.9/7.51	C_2, 6_-H_2, 6_ in H units (H) C_2, 6_-H_2, 6_, *p*-coumaroylated substructures (*p*CA) C_*α*_-H_*α*_, *p*-coumaroylated substructures (*p*CA)
	115.0/6.24	C_*β*_-H_*β*_, *p*-coumaroylated substructures (*p*CA)

G: guaiacyl unit; S: syringyl unit; H: *p*-hydroxyphenyl unit; pCA: *p*-coumaric acid; FA: ferulic acid

**Fig. 3:**
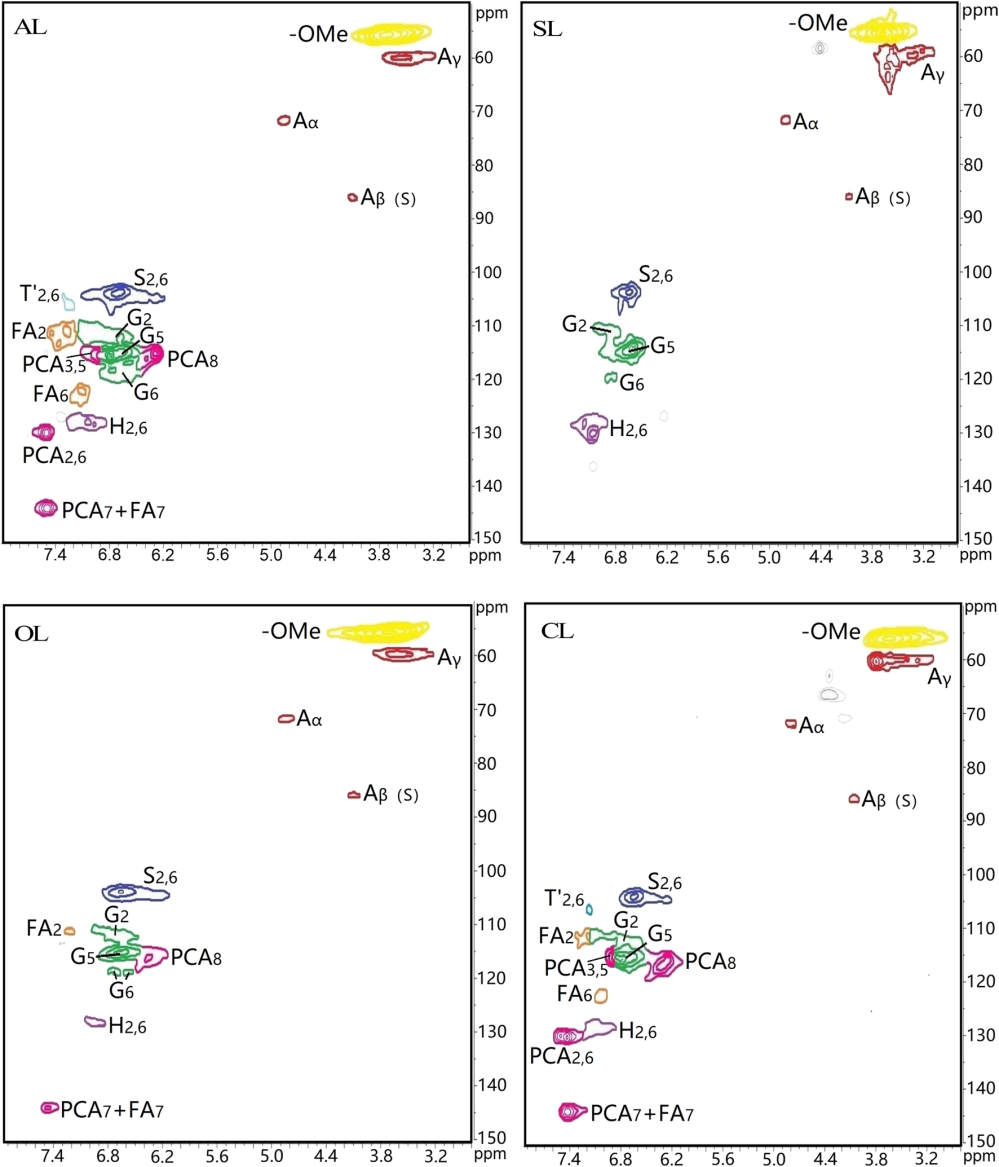
2D-HSQC NMR spectra of lignin samples

In the side-chain regions, the β-*O*-4′ aryl ether (A) and methoxyl groups (*δ*_C_/*δ*_H_ 55.4/3.72) are the most prominent signals observed in all spectra. The signals at *δ*_C_/*δ*_H_ 71.6/4.88 (*A*_*α*_), *δ*_C_/*δ*_H_ 82.8–85.7/4.13–4.46 (*A*_*β*_(G) and *A*_*β*_(S)), and *δ*_C_/*δ*_H_ 59.5–59.7/3.40–3.63 (*A*_*γ*_) belong to the C_α_-H_α_, C_β_-H_β_, and C_γ_-H_γ_ correlations of the β-*O*-4′ ether substructures, respectively. As shown in Fig. 3, the corresponding correlations of *A*_*α*_ and *A*_*β*_ were still detected and no changes occurred in all samples. However, it was interesting that by comparison with AL, three modified lignins exhibited the absorption of intense signals at *δ*_C_/*δ*_H_ 59.5–63.5.7/3.40–3.83 (*A*_*γ*_). This phenomenon suggested that the AL has been modified at the acylated γ-carbon in β-*O*-4′ aryl ether linkages of the side chains. And β-β′, β-1′and β-5′ substructures were not also observed in the spectra of modified lignin, suggesting that the surface modification of lignin could not cause the condensation between lignin units. Aromatic regions of the HSQC spectra can give basic correlation signals of the *p*-hydroxyphenyl (H), guaiacyl (G), and syringyl (S) lignin units. Specifically, the C_2,6_-H_2,6_ correlations signals of S and C_α_-oxidized S (T′) units were shown at *δ*_C_/*δ*_H_ 103.8/6.69 and *δ*_C_/*δ*_H_ 106.4/7.31. In Fig. 3, the signal intensity of C_2,6_-H_2,6_ in S unit tended to reduce in the modified samples compared to that of the control AL, indicating a decrease of S units in the surface of modified lignins, which was due to the cleavage of β-*O*-4 linkage. Similarly, the C_5_-H_5_, C_2_-H_2_, and C_6_-H_6_ correlations in G units, at *δ*_C_/*δ*_H_ 115.1/6.72, *δ*_C_/*δ*_H_ 111.0/7.02, and *δ*_C_/*δ*_H_ 119.0/6.77, respectively, were observed in very low intensities for three modified lignins. It is worth mentioning that *p*-hydroxyphenyl (H) units exhibited the strong signal for C_2,6_-H_2,6_ at *δ*_C_/*δ*_H_ 127.5/7.23 in others lignin, while it was weak in OL due to that oxidation treatment can result in less H units than that of other lignin samples. The above findings to aromatic ring units of lignin are consistent with FTIR analyses. In addition, the C_2_-H_2_, C_6_-H_6_, and C_7_-H_7_ correlations signals of ferulic acid (FA) were clearly observed at *δ*_C_/*δ*_H_ 110.8/7.35, 123.1/7.16, and 144.4/7.42 in the spectra. Alkali lignin showed a higher proportion of FA, but the intensity of FA signals could be weakened after the oxidized and carboxylic reaction, and it disappeared after sulfonated reaction. Simultaneously, the relatively strong signals at the *δ*_C_/*δ*_H_ 129.8/7.52, 143.9/7.51, and 115.0/6.24, corresponding to *p*-coumarates (pCA) were presented in all lignin samples except for SL; however, in comparison with AL, pCA units of oxidized (OL) and carboxylic lignin (CL) samples showed a slightly decrease. It had suggested that *p*-coumaric and acid ferulic acid are associated with the lignin monomer through either C–C or ether linkages and the carboxylic groups were not in free but in ester form [28–31]. This implied that pCA and FA in AL could be partly or completely hydrolyzed in the modification, and these groups could be broken into shorter side-chain products, such as vinyl phenols [32].

### Surface properties of lignin preparations

The XPS analysis can provide some knowledge about the elemental composition and functional group abundances on the near-surface regions of materials [33, 34]. As seen in Table 4, carbon and oxygen were the predominant elements detected on the all samples. Besides the two elements, the SL obtained from the sulfonation reaction contains sodium (7.17%) and sulfur (4.46%) and CL from carboxylation reaction has sodium (3.09%) and chlorinum (1.83%) element on the surface. As expected, the O/C ratios of SL, OL, and CL were 0.43, 0.35, and 0.38, respectively, and distinctively higher than that of AL, 0.28, suggesting that the surfaces of particles could have been altered after modification reaction. This lead to the conclusion that oxygen element could appear as free or esterified hydroxyl groups and free or esterified carboxyl groups in modified lignins. Moreover, XPS measurement also can give the information on functional groups of the sample surface. Especially, the abundance of C–C and C–H bonds (284.6 eV) decreased from 56.57% for AL to 54.90% for CL, 52.58% for OL and 38.29% for SL, suggesting that the decrease of condensation degree in lignin after the modification reactions. Quantities of –C–O– bonds on the surface of CL and SL were 33.13 and 7.29%, respectively, which were lower than in AL (39.65%) and OL (39.84%). A significant increase of carbon atoms bonded with carbonyl or non-carbonyl oxygen (O–C=O, 289 eV) in modified samples, OL and CL, was also observed and the amount of O–C=O bonds on the surface of OL and CL were 1–2.17 times higher than that of AL. This will be beneficial to increase the hydrophilicity of modified lignins. Although the content of –C–O– and O–C=O bonds on SL surface was much lower than those of AL, the sulfonate groups in SL, incorporated by sulfonation reaction, were hydrophilic groups, thus the surface of SL had hydrophilicity.

**Table 4:** Elemental composition and functional groups based on the C1s peaks by XPS analysis

Samples	Element composition (%)					Surface chemical groups (%)		
	C	O	Na	S/CL	O/C ratio	C–C/C–H (284.6, e V)	C–O (286.6, e V)	O–C=O (289, e V)
AL	78.14	21.86	/	/	0.28	56.57	39.65	3.78
SL	61.75	26.62	7.17	4.46 (S)	0.43	38.29	7.29	2.45
OL	74.27	25.73	/	/	0.35	52.58	39.84	7.58
CL	69.05	26.06	3.09	1.83 (CL)	0.38	54.90	33.13	11.98

The total concentration of free phenolic groups in all lignins using the F–C method is listed in Table 5. The phenolic group content in SL, OL, and CL were estimated to be 0.56, 0.91, and 0.21 mmol/g, respectively. By comparison with AL of 1.09 mmol/g, the modified lignins showed lower Ph–OH content, indicating the sulfonated, oxidized, and carboxylation modification could contribute to the decrease of phenolic group content in lignin preparations. The content of free phenolic groups in modified lignins decreased by about 17–91% compared with AL. It suggested that the AL fractions were obtained by alkali extraction under sharp condition, which can produce high phenolic content by the cleavage of ether bonds. The measurements of contact angle against water were carried out to reveal the hydrophobic character of lignin surface. As can be seen in Table 5, the contact angles decreased very quickly with the introduction of hydrophilic functional groups and units. The AL exhibited a contact angle with water of 98.7°, while those of SL, OL, and CL were 29.4°, 29.5°, and 38.2°, respectively, suggesting that three modified lignins had stronger relative hydrophilicity and permittivity. The surface structural differences between AL and modified lignins can account for this result, because there was a higher increase of *p*-coumaric, acid ferulic acid, sulfonate groups, and aliphatic COOR, and a decrease of condensation degree and S-lignin, G-lignin unit in the modified lignins. The results were further indication that the sulfonated, oxidized, and carboxylation modification of lignin could be used to adjust lignin chemical functionality and surface hydrophilicity. Zeta potential values of all lignin samples were measured and are compared in Table 5. The zeta potential of AL was determined to be 3.5 mV, suggesting that not only no repulsion existed, but there was attractive force between negative charged proteins and AL. However, the zeta potential values of three modified lignins was negative, − 21.5 mV for OL, − 27.3 mV for CL, and − 45.9 mV for SL, respectively. And the SL displayed 0.7–1.1 times higher zeta potential than CL and OL due to sulfonic groups. So, the higher negative zeta potential of SL, OL, and CL would cause stronger electrostatic repulsion between modified lignins and negative charged proteins in cellulase enzymes.

**Table 5:** Water contact angles, Zeta potentials and phenolic group content in lignin samples

Samples	Zeta potential (mV)	Contact angle (°)	Phenolic group content (mmol/g)
AL	3.5	98.7	1.09
SL	− 45.9	29.4	0.56
OL	− 21.5	29.5	0.91
CL	− 27.3	38.2	0.213

### Effects of alkaline lignin modification on nonproductive adsorption of cellulase

**Fig. 4:**
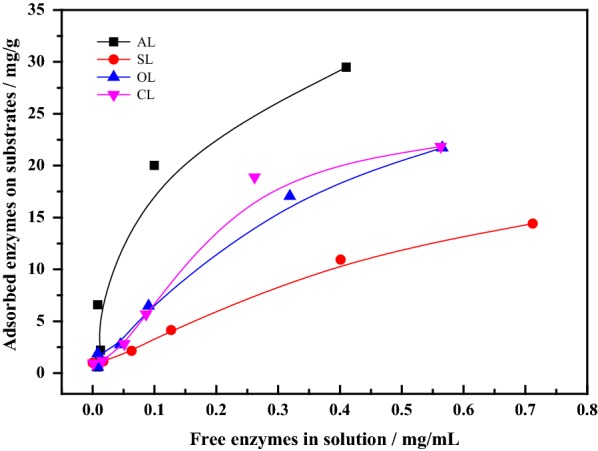
Cellulase enzyme adsorption on modified lignins. Lignins and enzyme cellulases (0.01–2 mg/mL) were incubated in 0.05 M citrate buffer at 4 °C and 150 rpm for 3 h to reach equilibrium

To examine how the modification in AL interfered the cellulase–lignin interaction, the cellulase adsorption on lignin samples and Langmuir adsorption isotherms of cellulase enzymes were analyzed and are summarized in Fig. 4 and Table 6. The AL had the highest affinity for cellulase (5.92 mL/mg protein), whereas SL had the lowest affinity (1.16 mL/mg protein) for cellulase, followed by OL (2.25 mL/mg protein) and CL (2.72 mL/mg protein). Similarly, the distribution coefficient (*R* = *K* × *Γ*_m_) of cellulase on AL that represented the binding strength of lignin to cellulase enzymes was 0.253 L/g lignin, which was two to threefolds higher than those on SL (*R* = 0.083 L/g), OL (*R* = 0.089 L/g) and CL (*R* = 0.104 L/g). It was noticed that the binding strength of AL (*R* = 0.253 L/g) was even higher than that of Avicel (R = 0.133 L/g), suggesting that AL would get more chance to adsorb cellulase enzyme than cellulose. Consequently, after modification reaction, the maximum adsorption capacity of lignin toward enzymes was significantly reduced. Specifically, AL (*Γ*_m_ = 42.78 mg/g lignin) had stronger adsorption capacity, which can adsorb about 7.9–24% more protein than modified lignins. And the maximum adsorption capacity of modified lignins decreased in the order OL > CL > SL, among which sulfonic acid groups caused greater decrease in the binding strength than the oxidized and carboxylate groups. This was probably because the introduction of sulfonic groups could consume more phenolic hydroxyls and bring more negative charges in lignin moieties to some extent.

**Table 6:** Langmuir adsorption isotherm parameters from enzyme adsorption on substrates

Substrates	*Γ*_m_ (mg/g)	*K* (mL/mg)	*R* (L/g)
Avicel	48.57	2.74	0.133
AL	42.78	5.92	0.253
SL	32.43	1.16	0.038
OL	39.41	2.25	0.089
CL	38.12	2.72	0.104

We believe that the decrease of cellulase–lignin interaction was related to the structure and physicochemical properties of modified lignins. As well known, the hydrophobic, electrostatic, and hydrogen-bonding interactions could mediate the nonproductive adsorption of cellulase onto lignin. Alkali lignin had a contact angle of 98.7° and the binding strength of 0.253 L/g lignin. The modification led to a decrease in contact angle of lignin by 61–70%, due to the drop in the degree of condensation and lignin subunits, such as syringyl (S) and guaiacyl (H), and the more exposed carboxylic groups. This can make the lignin more hydrophilic, thereby decreasing the nonproductive binding for cellulase. In addition, the functional groups introduced into AL also had a much greater impact on the surface charge of lignin moieties and the formation of hydrogen bonds. According to XPS analysis (O–C=O, 289 eV) and C13 NMR (178–168 ppm), a low content of carboxylic groups was introduced to the CL and OL surface, and the abundant of sulfonic acid groups also was incorporated into SL in the FTIR (S=O stretching vibration, 1043 cm^−1^), thus the zeta potential values of modified lignin were increased from 3.5 eV (AL) to − 21.5 eV (OL), − 27.3 eV (CL) and − 45.9 eV (SL), which increased the electrostatic repulsion between enzyme and lignin and prevented the nonproductive binding. And with the consumption of phenolic hydroxyl groups in the sulfonation, oxidation, and carboxylation, the content of phenolic hydroxyls decreased by 49, 17, and 80%, respectively, comparing to AL. This meant the decrease of binding strength between cellulase and modified lignins, because the cellulase adsorption on lignin correlated positively with phenolic hydroxyl content [15, 16]. As a result, the hydrophobic, electrostatic and hydrogen-bonding interactions together had exerted synergistic effects upon weakening the nonproductive adsorption of cellulase onto modified lignins, led to the affinity constants and binding strengths decrease by 59–85% than AL, which would stimulate the enzymatic digestion of cellulose.

### Effects of alkaline lignin modification on glucose yield and cellulase activity in enzymatic saccharification of Avicel

**Fig. 5:**
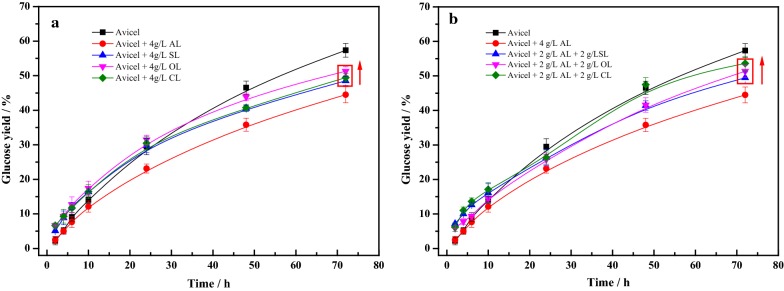
Effects of modified lignins on glucose yield in enzyme saccharification of Avicel. **a** Enzymatic saccharification of Avicel with lignin loading of 4 g/L; **b** enzymatic saccatification of Avicel with mixture of AL (2 g/L) and modified lignins (2 g/L). Enzymatic saccharification was conducted at 50 °C and 150 rpm in the 0.05 M sodium acetate buffer (pH 4.8) for 72 h, with the cellulase (UTA-8) loading of 10 FPU/g glucan

As showed in Fig. 5, all lignin preparations had a great inhibitory effect on the 72 h glucose yield of Avicel, which were consistent with the above the enzymatic adsorption data. The enzymatic saccharification of Avicel with lignin loading of 4 g/L was evaluated in Fig. 5a. For the hydrolysis of pure cellulose, Avicel, the 72 h glucose yield was 57.4%. As can be seen in Fig. 5a, although all of lignin preparations displayed the inhibition compared with pure cellulose, the addition of modified lignins obviously reduced the negative effect on enzymatic hydrolysis. The 72 h glucan conversion with addition of AL was 44.5%, while the glucan conversion at 72 h with addition of SL, OL, and CL were 48.5, 51.3, and 49.4%, respectively. The addition of SL, OL and CL enhanced the 72 h hydrolysis yield of Avicel by 8–15.3% than that of Avicel with AL. Remarkably, it was observed that OL from oxidized modification showed much higher stimulation than other two modified lignins. In addition, the enzymatic hydrolysis of Avicel with mixture of AL (2 g/L) and modified lignins (2 g/L) are also assessed in Fig. 5b. As expected, the mixture with AL and modified lignins also weakened the inhibitory effect of AL alone on enzymatic hydrolysis under the same content of lignin. Particularly, addition of AL and CL had the highest glucose yield (53.6%), followed by the mixture of AL and CL (51.36%) and that of AL and SL (49.5%), increased by 11.2–20.4% than AL of 4 g/L alone (44.5%).

**Fig. 6:**
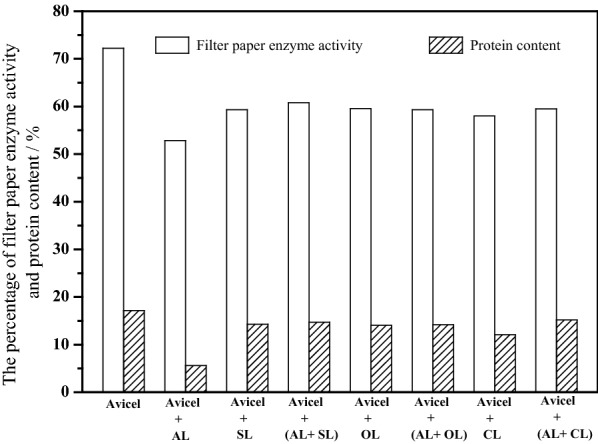
Effect of modified lignins on relative enzyme activity and protein content at 72 h in enzymatic saccharification of cellulose

Besides, the effect of modified lignins on the cellulase activity was assessed by measuring the free enzyme activity and the protein concentration of supernatant in the 72 h of hydrolysis process. As shown in Fig. 6, after 72 h incubation at pH 4.8 and 50 °C, filter paper enzyme activity and protein content at the 72 h hydrolysis of Avicel alone retained 72.2 and 17.1% of its initial activity, while that of Avicel with 4 g/L of AL decreased to 52.8 and 5.6% of its starting activity, indicating that 27% cellulase activities and 67% protein were adsorbed to AL or inactivated by the nonproductive adsorption of cellulase onto lignins. However, the inhibitory effect of lignin on enzyme activity and protein content was greatly reduced, when AL were replaced with modified lignins. This improvement made enzyme activity percentage increased up to 59–61%, and protein content percentage up to 14–15%, which was 12–16% and 1.5–1.7 times higher than cellulase activity and protein content of AL alone in the 72 h of hydrolysis process, respectively. The results suggested that the modified lignins did not lead to the decrease of cellulase activity in supernatant, on the contrary, can increase free cellulase enzymes and decrease cellulase activity loss than the presence of AL, which can reduce the negative effect of lignin preparations on enzymatic digestibility.

### Effects of alkali pretreatment and post-treatment on glucose and xylose release in enzymatic hydrolysis of bamboo

**Fig. 7:**
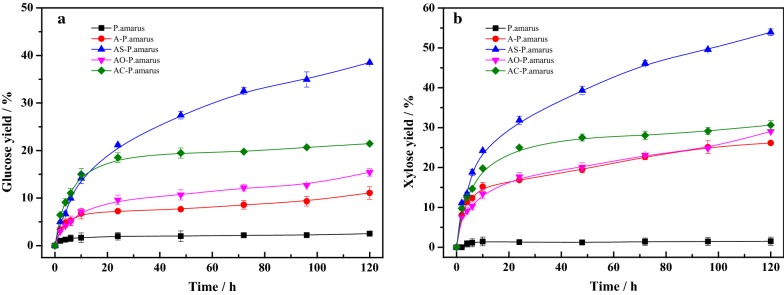
The enzymatic hydrolysis efficiency of the combination with alkali pretreatment and sulfonation, oxidation, and carboxylation post-treatment in *P. amarus*. **a** Glucose yield. **b** Xylose yield

To verify whether lignin modifications as post-treatment technologies can work on lignocellulosic biomass, bamboo (*P. amarus*) was extracted with 10% (w/w) sodium hydroxide at 70 °C for 3 h, followed by sulfonation, oxidation and carboxylation post-treatment. As seen in Fig. 7, for each post-pretreatment, the increase of glucose and xylose release was observed in the hydrolysis process. Untreated *P. amarus* almost was not degraded by enzymatic cellulase due to the strong recalcitrance. The 5 days hydrolysis of alkali pretreated *P. amarus* (A-*P. amarus*) only obtained the glucose yield of 11.1%. However, when alkali pretreatment was followed by the sulfonation, oxidation and carboxylation post-pretreatment, glucose yield was increased to 38.5% (AS-*P. amarus*), 15.4% (AO-*P. amarus*), and 21.4% (AC-*P. amarus*), respectively. The corresponding xylose yield was also increased from 26.2 to 53.9, 29 and 30.9%, respectively. And the enhancing effects caused by sulfonation post-treatment were greater than oxidation and carboxylation post-pretreatment. The results suggested that the post-pretreatment by modifying lignin can enhance glucose and xylose release in the enzymatic hydrolysis of lignocellulosic biomass. We believe that the increase of glucose and xylose yield after post-treatment can most likely be attributed to the decrease of lignin inhibition on enzymatic hydrolysis. In addition, it is important that alkali pretreatment is still considered as the appropriate method for developing the combined pretreatment technologies for reducing lignin inhibition on enzymatic hydrolysis. Because alkali pretreatment can decrease the lignin content, the sulfonation, oxidation, and carboxylation post-treatment can reduce the nonproductive adsorption of cellulase on lignin, which could contribute to the decrease of lignin inhibition together.

It was well known that lignocellulosic substrates pretreated with thermochemical methods have been reported to require subsequent post-treatment to enhance enzymatic saccharification efficiency by decreasing the lignin content and modifying the lignin structure [35]. Many studies on post-treatment had focused on a drop in the lignin content of pretreated substrates with the objective of delignification. The delignification is costly for the existing thermochemical pretreatment technologies and could bring about polysaccharides loss, which may be worth exploring at a later date. We believed that post-treatment with the objective of modifying the lignin structure, especially under the mild conditions, would attract considerable attention, which could help to design a pretreatment scheme for reducing the negative effect of lignin on enzymatic hydrolysis.

## Conclusions

When the carboxylic, sulfonic acid, and oxidative groups were incorporated into AL, the decrease of hydrophobic and hydrogen-bonding interactions and the increase of electrostatic interactions between modified lignins and enzyme cellulase were observed. Langmuir adsorption isotherms also proved that modified lignin had a lower binding ability to the cellulase enzyme than AL. Therefore, the 72 h glucose yields of Avicel with modified lignins increased from 44.5% for AL to 48.5% for SL, 49.4% for CL, and 51.3% for OL, suggesting the drop of lignin inhibition on enzymatic digestion. Modified lignins made enzyme activity percentage increased by 12–16% at the 72 h of hydrolysis process than AL. In addition, we detected that combination of alkali pretreatment and sulfonation, oxidation and carboxylation post-treatment enhanced glucose yield of *P. amarus* by 28–71.2% and xylose yield by 15.2–51.4%. This study is helpful to design a pretreatment scheme involving the sequential coupling of pretreatment methods and post-treatment with the objective of reducing the lignin inhibition on enzymatic hydrolysis.

## Methods

### Chemicals and materials

Alkali Lignin, a mixture of different herbaceous plants, such as corn stover, bamboo and straw, were supplied by Shanghai Yuanye Bio-Technology Co., Ltd (Shanghai), which was obtained by NaOH pretreatment and then purified by an acidulation precipitation method. The chemical composition of AL was as follows (dry weight basis): 90.9% acid insoluble lignin, 3.67% acid soluble lignin, 0.1% glucan, 0.05% xylan, 2.19% ash, and 2.79% others. Microcrystalline cellulose (Avicel PH-101, ~ 50 μm particle size) was purchased from Sigma-Aldrich (Shanghai). The bamboo (*Pleioblastus amarus, P. amarus)* was obtained from Yunnan, China and its glucan, xylan, and lignin contents were 41.7, 15.7 and 37.5%, respectively. Cellulase (UTA-8) from *Trichoderma reesei* with the filter paper activity of 100 FPU/mL and the β-glucosidase activity of 71 IU/mL, was kindly donated by Youteer Biochemical Co., Ltd. (Hunan) and used in subsequent enzymatic saccharification of cellulose. Celluclast 1.5 L (Sigma 2730) from Sigma-Aldrich (Shanghai) exhibited the protein content of 40 mg/mL and was employed in experiments of cellulase adsorption.

### Lignin modification

Three modified lignins were prepared from AL. Sulfonated Lignin (SL) was obtained by treating 10.0 g AL with 30 mL NaOH solution (0.8 M) and 1.5 mL formaldehyde in 70 °C for 90 min. Sodium sulfite of 2 g was mixed with the above solution to carry out sulfonation reaction at 95 °C and 3 h [36]. Oxidized Lignin (OL) was prepared based on the Fenton oxidation reaction reported in previous literature [37, 38]. In brief, AL (5 g) was dissolved in 100 mL deionized water with a little NaOH at 50 °C and the pH was adjusted to 3–5. The 30% H_2_O_2_ of 2 mL/g lignin and FeSO_4_ (16 mM) were added in the solution and the mixture was reacted at room temperature (22 ± 1 °C) and 80 rpm in a water bath for 24 h. Carboxylated Lignin (CL) was prepared through the reaction between hydroxyl groups and sodium chloroacetate, as described previously [39]. The solution of AL was mixed with sodium chloroacetate of 4 mol/L at the ratio of 2:1. The reaction was conducted at 70 °C for 90 min. After the reaction, the pH of solution was adjusted to precipitate lignin, and the sample was collected by centrifugation, washed with warm water and dried under vacuum.

### Surface and structural analysis of lignin preparations

The lignin preparations were analyzed using a 710 FTIR spectrophotometer (Nicolet, USA). The ^13^C and HSQC NMR spectra of lignins were acquired with a Bruker AVIII 400 MHz spectrometer. The sample (80 mg) was placed in NMR tubes with 0.5 mL dimethyl sulfoxide-d6. 2D HSQC spectra were recorded on the same spectrometer using 128 scanning time, a 2.6-s delay time between transients, and a 1.5-s relaxation time. X-ray photoelectron spectroscopy (XPS) analyses was conducted on an Escalab 250Xi spectrometer (Thermo Fisher Scientific Inc., US), with non-monochromatic Al *K* alph (X-ray-voltage 15 kV, 300 W, X-ray energy 1486.8 eV), under a high vacuum of 2 × 10^−9^ mbar and at room temperature. The content of free phenolic group in lignin samples were estimated by the Folin–Ciocalteu (F–C) method using phenol as standard [24, 40, 41]. The contact angle was measured using OCA-20 contact angle meter (Dataphyscis Inc., Germany) at room temperature (22 ± 1 °C). The average contact angle (*θ*) values were obtained from measurements at three points in each lignin surface. The measurements of Zeta potentials were analyzed using a Malvern Zetasizer Nano ZS instrument. Lignin preparations (0.01%, w/w) were incubated in 0.05 M citrate buffer (pH 4.8) for 1 h, followed by determination of zeta potential. The reported result of each sample was the average of three trials.

### Enzyme adsorption isotherm of lignin preparations

The adsorption isotherm of enzyme on lignin preparations was studied with different concentration cellulases from 0.01 to 2 mg/mL [42]. Lignins and enzymes were incubated in 0.05 M citrate buffer at 4 °C under shaking (150 rpm) for 3 h to reach equilibrium. The concentration of free proteins in the solution was monitored using Bradford method. The adsorbed enzyme was calculated by subtracting the amount of free proteins from the total initial protein amount. All adsorption reactions were run in parallel. Maximum adsorption capacity (*Γ*_m_, mg/g lignin) and affinity constant were estimated by nonlinear regression of adsorption data according to the Langmuir isotherm equation (*Γ* = *Γ*_m_*KC*/(1 + *KC*)), where *Γ* is the amount of adsorbed enzyme (mg/g substrate), *C* the amount of free enzyme in solution (mg/mL) and *K* the Langmuir constant (mL/mg enzyme). And the distribution coefficient (*R* = *Γ*_m_ × *K* L/g), represented binding strength, was also analyzed.

### Enzymatic hydrolysis

#### Enzymatic hydrolysis of Avicel

Enzymatic hydrolysis was conducted at 50 °C and 150 rpm in the 0.05 M sodium acetate buffer (pH 4.8) for 72 h, with a solid substrate loading of 2% (w/v) Avicel. The dosage of cellulase (UTA-8) was 10 FPU/g glucan. To investigate the effects of lignin compounds (AL, SL, OL, and CL) on enzymatic hydrolysis, the 4 g/L lignin sample (AL, SL, OL, and CL, respectively) or the mixture of 2 g/L AL with 2 g/L (SL, OL, and CL) were added into pure cellulose, respectively, prior to the addition of cellulase. Enzymatic saccharification with addition of AL only was used as the control. Aliquots (0.2 mL) were withdrawn from the supernatant at 2, 4, 6, 10, 24, 48 and 72 h of hydrolysis. Quantitative analyses of glucose were measured by an Agilent 1260 series HPLC equipped with Aminex HPX-87H column at 60 °C column temperature, using 5 mM H_2_SO_4_ as eluent at the flow rate of 0.6 mL min^−1^, as described before. The glucose yield was defined as the percentage of the glucose content in hydrolysate based on the theoretical glucose available in the cellulose. And protein concentration in supernatant was determined by Bradford assay, and calculated based on the total protein concentration. The relative enzyme activity at 72 h of enzymatic hydrolysis was also measured using the filter paper assay [43].

#### Enzymatic hydrolysis of lignocellulosic biomass

*P. amarus* was extracted at 70 °C for 3 h with 10% NaOH (w/w) based on the dry weight of biomass, in a solid to liquid ratio of 1:10 (w/w). After washing with water, the solid residue was subjected to sulfonation, oxidation and carboxylation post-treatment; the post-treatment condition was the same as that of the AL. The effects of combination of alkali pretreatment and post-treatment were evaluated based on the subsequent glucose and xylose release in the hydrolysate. Enzymatic hydrolysis of pretreated substrates was conducted at 50 °C, pH 4.8 and 150 rpm for 5 days, with a substrate loading of 5% (w/v) and a cellulase (UTA-8) loading of 20 FPU/g glucan. The glucose and xylose yield were defined as weight percent yield of glucose and xylose with respect to the total glucan and xylan in the bamboo biomass. The compositions of alkali pretreated *P. amarus* (A-*P. amarus*) were 51.8% glucan, 17.5% xylan, and 35.1% lignin. The combination with alkali pretreatment and sulfonation, oxidation and carboxylation post-treatment of *P. amarus* was defined as AS-*P. amarus* and AO-*P. amarus* and AC-*P. amarus*, respectively. After post-treatment, AS-*P. amarus* contained 50.9% glucan, 13.1% xylan, and 23.4% lignin; and AO-*P. amarus* 54.4% glucan, 15% xylan, and 27.1% lignin; and AC-*P. amarus* 60.9% glucan, 14.8% xylan, and 21.6% lignin, respectively.

## Abbreviations

SL: Sulfonated Lignin
OL: Oxidized Lignin
CL: Carboxylated Lignin
AL: Alkali Lignin
A-*P. amarus*: *P. amarus* pretreated with alkali
AS-*P. amarus*: *P. amarus* pretreated with alkali and sulfonation post-pretreatment
AO-*P. amarus*: *P. amarus* pretreated with alkali and oxidation post-pretreatment
AC-*P. amarus*: *P. amarus* pretreated with alkali and carboxylation post-pretreatment
S-lignin: syringyl lignin
H-lignin: *p*-hydroxyphenyl lignin
G-lignin: guaiacyl lignin
FA: ferulic acid
pCA: *p*-coumarates
XPS: X-ray photoelectron spectroscopy
HPLC: High-performance liquid chromatography
FTIR: Fourier transform infrared spectroscopy
13C NMR: ^13^C nuclear magnetic resonance
HSQC spectra: Heteronuclear single-quantum coherence spectra
F–C method: Folin–Ciocalteu method
